# "Care is not care if it isn't person‐centred": A content analysis of how Person‐Centred Care is expressed on Twitter

**DOI:** 10.1111/hex.13199

**Published:** 2021-01-28

**Authors:** Cornelia van Diepen, Axel Wolf

**Affiliations:** ^1^ Institute of Health and Care Sciences Sahlgrenska Academy University of Gothenburg Gothenburg Sweden; ^2^ Erasmus School of Health Policy & Management Erasmus University Rotterdam Rotterdam Netherlands; ^3^ Centre for Person Centred Care University of Gothenburg Gothenburg Sweden

**Keywords:** content analysis, Person‐Centred Care, tweets, Twitter

## Abstract

**Background:**

Person‐Centred Care (PCC) has been the subject of growing interest in recent decades. Even though there is no conceptual consensus regarding PCC, many health‐care institutions have implemented elements into their care.

**Objective:**

This study aimed to investigate the PCC topics presented by different stakeholder groups on Twitter and to explore the perceptions of PCC within the content of the tweets.

**Method:**

Tweets with mentions of PCC in various translations were collected through a Twitter Application Programming Interface in October 2019. The tweets were analysed using quantitative and qualitative content analysis.

**Results:**

Five stakeholder groups and ten topics were identified within 1540 tweets. The results showed that the PCC content focused on providing information and opinions rather than expressing experiences of PCC in practice. Qualitative content analysis of 428 selected tweets revealed content on a vision that all care should be person‐centred but that the realization of that vision was more complicated.

**Conclusions:**

Twitter has shown to be a quick and non‐intrusive data collection tool for uncovering stakeholders' expressions concerning PCC. The PCC content revealed that stakeholders feel a need to 'educate' others about their perception of PCC when experiences and real‐life applications are missing. More action should be taken for the implementation of PCC rather than circulating PCC vision without operationalization in care.

**Public Contribution:**

The public provided the data through their posts on Twitter, and it is their perception of PCC that is studied here.

## 1 INTRODUCTION

Since 1960, more emphasis has been aimed at the patients' active role within health care to increase the patients' involvement in care and treatment, by taking patients' voices, experiences and demands seriously into consideration in health‐care provision.^1^ Health‐care professionals, patient organizations and policymakers increasingly aspire to Person‐Centred Care (PCC) to optimize cost containment and quality of care.^2^ PCC is directed at improving the health and recovery process of patients and improving the work environment of health‐care professionals by forming a partnership between the patient/relatives and the health‐care professionals. PCC constitutes an approach to health care based on ethical principles, which are summarized in three core components: inclusion of patients' narrative, co‐creating a health plan and monitoring that health plan.^1^ Evidence from research studies regarding PPC outcomes is substantial.^3^

Nevertheless, real‐world implementation of PCC is lacking, which could be because its conceptualization and perception are still poorly understood.^4^, ^5^ For example, there is an array of different concepts and terms for PCC that are often used interchangeably, for example Patient‐Centred Care, Individual‐Focused Care and Person‐Centred Care.^5^, ^6^, ^7^ Also within PCC itself, the range of definitions and practices claiming to be person‐centred is broad, and the need for stringency within the concept of PCC has been highlighted.^5^, ^8^, ^9^, ^10^, ^11^, ^12^ A recent review of PCC identified three pillars that are not mutually exclusive, yet still different: PCC as emphasizing personhood, PCC as a partnership and PCC as an overarching holistic approach that comprises many different activities, principles and enablers such as self‐care and decision making.^13^ Hence, while PCC seems to be a crucial component in qualitative health care, it appears to be both perceived and experienced differently depending on, for example, different stakeholders and context.

Twitter, as a social network site, is well‐positioned to explore these perceptions and experiences because it is rapidly becoming a key resource for opinion and policymakers, patient organizations and industry, as well as public health surveillance.^14^ This social network site contains vast amounts of freely available, user‐generated microblogs and reflects unprompted opinions on public health matters. Twitter provides real‐time monitoring on public health topics in an efficient and automated manner. Eysenbach defines this monitoring as ‘Infoveillance’ which is ‘the science of distribution and determinants of information in an electronic medium, specifically the Internet, or in a population, with the ultimate aim to inform public health and public policy’.^14^ ^(p2)^ Infoveillance may have many benefits to the field of PCC research as it could provide new insights into the perceptions of PCC people find most important to share with their social network.

Twitter provides many single entries and hardly contains any context to the post. Therefore, most early work on Infoveillance used elaborate models to analyse patterns in the use of the Internet through automated queries.^14^, ^15^ However, the potential for applying social research methods has gained more attention in recent years as, although meagre, the microblogs contain useful information on the chosen topic and the Twitter user posting the tweet.^16^ The data provide a slice of the general population (with mostly affluent young users) and allows for many different voices to be included in the study without having to contact them individually.^17^ Tweeting can be considered a performative platform to self‐identify.^18^, ^19^ By interacting or simply spreading posts, a performance of self is provided for an imagined audience.^20^ Therefore, the analysis of the PCC contents of the tweets offered both a better understanding of how PCC is expressed by stakeholders and an indication of why the tweet was made.

The purpose of this study was twofold: to investigate the PCC topics presented by different stakeholder groups on Twitter and to explore the perceptions of PCC within the content of the tweets.

## 2 METHODS

### 2.1 Data collection

The PCC‐related Twitter search was conducted in the Twitter Application Programming Interface (API) called Mozdeh,^21^ which is one of the many freely available programmes. The search terms consisted of 'Person‐Centred Care' translations from 16 European languages (Supplementary Table 1). These search terms were compiled from the Cost Action 15222 COST CARES network in which stakeholders from each country offered their local term for PCC.

The API collected PCC‐related tweets posted between 1 October and 31 October 2019. Besides the PCC‐related content of the tweets, the data derived from the API encompassed an ID‐number, a timestamp, name, username and language.

**FIGURE 1:**
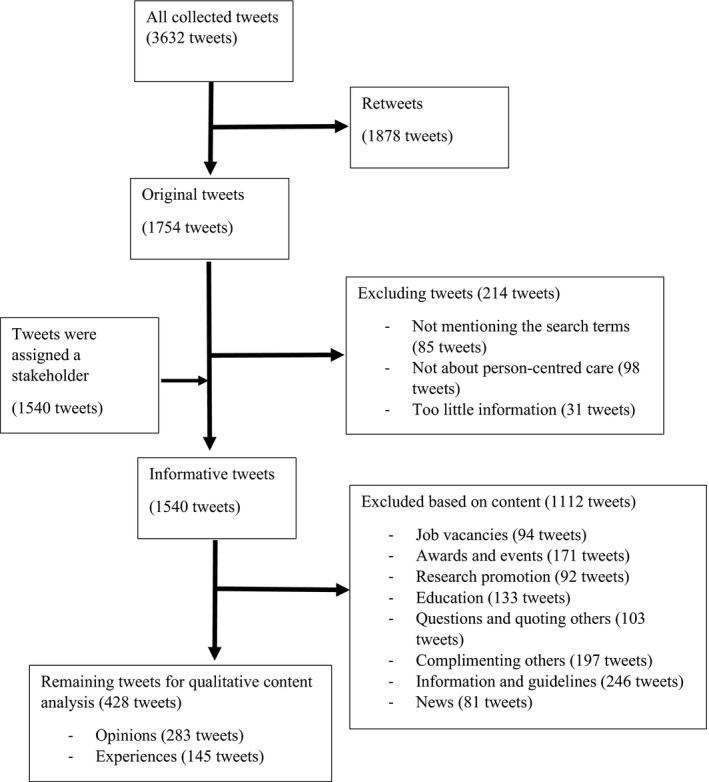
Exclusion diagram

The API generated 3 632 tweets consisting of 1 231 original tweets, 1 878 retweets and 523 replies. Only the original tweets and replies were used in this study and accumulated to a sample of 1 754 tweets for the content analysis. The flow chart (Figure 1) presents the steps of this study.

### 2.2 Content analysis

For the content of the tweets, we applied the content analysis method as described by Graneheim & Lundman.^22^ The tweet's content was read line by line and coded by the first author, whereby meaning units were created and categorized. All tweets could only fit one category to avoid over over‐calculation of the number of tweets. When the tweets did not contain the search term in the order of the concept (e.g. ‘Communities of care centred around the patient/person’), not refer to the concept of Person‐Centred Care (e.g. ‘I don’t care for this self‐centred person’) or contained no more than the concept (e.g. ‘Person‐centred care?’), they were coded as 'other' and not included in the results.

**FIGURE 2:**
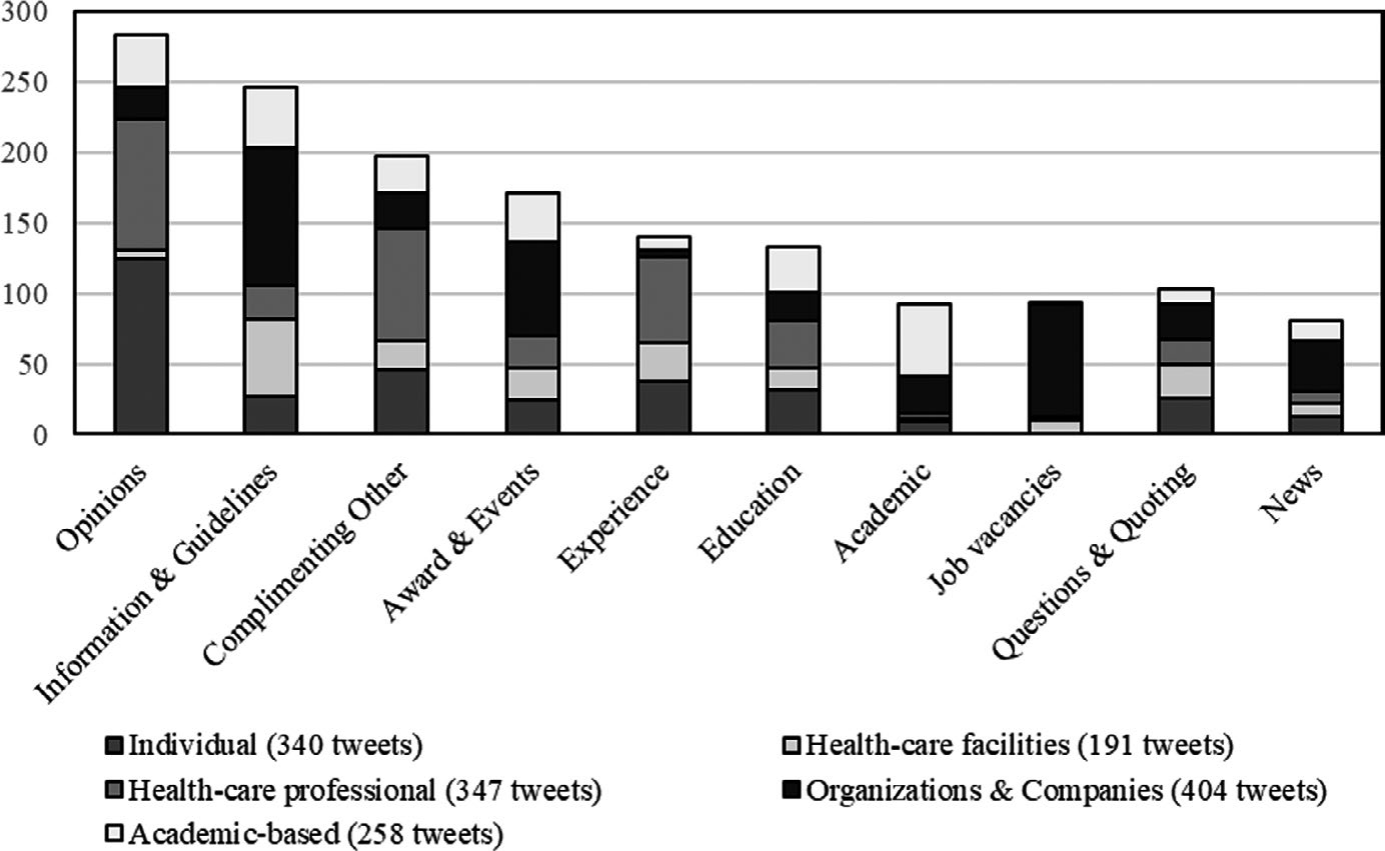
Bar chart of the topic and stakeholder groups of the tweets mentioning Person‐Centred Care.

For the quantitative analysis, we started by allocating the author posting the tweet to stakeholder groups with 'individual' as standard. This allocation was based on the name, username or explicit content of the tweet. If there was no clear indication of the stakeholder group, the author remained an 'individual'. This group of 'individuals' could contain a wide variety of Twitter users. We also grouped the tweets into the common topics, see Figure 2.

In the qualitative content analysis, the tweets were further coded to interpret the meaning within their topic. These topics can be understood as the latent content of the text.^22^ ^(p107)^ The purpose of this analysis was to explore the perceptions of PCC expressed in the tweets. Consequently, the different topics were scrutinized for content that encompassed a perception of PCC. Two topics (i.e. Opinions & Experiences) were eligible for the qualitative content analysis into the perceptions and experiences of PCC on Twitter. An example of the coding scheme is shown in Table 1.

**TABLE 1:** Example of the Coding scheme

Tweet	Author	Meaning unit	Sub‐Category	Category
*‘Providing Person centered care is the way to humanise our patients #patientexperience’*	Individual	Opinion	Vision of PCC	Care should be PCC
*‘My experience with advanced care plans is that they are always person centred and empower the person during their treatment as well as those delivering care and treatment, and also support a dignified death #WeLDNs’*	Health‐care professional	Experience	Facilitating PCC	
*‘@’health facility', I thought he was doing wonderfully xxx my son has cerebral palsy too and does not let his disability hold him back, much like how Ethan comes across. My son has a fantastic physio whom he sees at the “healthcare facility” which is another fabulous place person Centred Care xx’*	Individual	Experience	Experiencing PCC	
*‘rules and procedures can get in the way of person Centred Care’*	Health‐care facility	Opinion	PCC is not that simple	Challenging to realize
*‘It should be last person centered Care because unfortunately the reality is it is the last person left. Reality is true demand out does the supply of services needed for the person in question. A lot of the work/fight for services falls upon the carer be it family or employed’*	Health‐care professional	Opinion	Lack of resources	
*‘@### Most individuals—whether kids or adults, leaving state care or state institutions, not getting the appropriate person‐centered aftercare and therefore face stigma, accelerated health problems and loneliness. Putting them at risk’*.	Individual	Opinion	Lack of access	

### 2.3 Ethical considerations

Twitter is a public platform in which the data are freely available. Upon signing up for Twitter, there is a built‐in application that acquires consent for the use of the individuals' feed by third parties. Anyone can access and use the tweets provided by the individual on the Twitter social network site, which prompts many studies to refrain from mentioning ethics at all.^15^

Several papers did discuss the potential for social media, such as Twitter, to be damaging to the individual and presented guidelines to overcome such difficulties.^23^, ^24^, ^25^ Following these guidelines surpasses the need to inform the individuals of their participation and to receive consent.^26^ The tweets were directly copied from the API programme to preserve the expression of the author, which makes it possible for the tweets to be found when searched for on Twitter, but ensures transparency of the used data. Yet, this paper followed the general guidelines for preventing direct harm or identification by replacing all usernames and in‐text references by hashtags. Alternative references (e.g. @‘ambulance service') were given when it was vital for the understanding of the tweet.

## 3 RESULTS

### 3.1 Descriptive quantitative analysis

The total number of tweets in the analysis was 1 754 original tweets and replies made in eight different languages (i.e. English (1552), Dutch (72), Swedish (30), Spanish (18), Danish (3), French (2), Arabic (1), German (1) and Undefined (64)). The authors have language proficiency of English, Dutch, Swedish, Danish and German. The tweets in Spanish, French and Arabic tweets (n=21) were translated by Google Translate, and to improve accuracy, native speakers have checked these translations. The tweets with an undefined language had a majority of emoji's, abbreviations or 'slang' which made it impossible for the API programme to assign a language and for us to analyse objectively. As none of the tweets had a geolocation identifier, it is unclear from which country, region or city the tweets originated.

After removing those tweets not related to the concept of PCC or exhibiting too little information, the open coding of the 1540 remaining tweets shaped ten topics. We identified five groups of stakeholders within the tweets: individuals (i.e. unspecified Twitter users), health‐care facilities (directly providing care, e.g. hospitals and elderly care homes), health‐care professionals (e.g. doctors and nurses), organizations & companies (any enterprise connected to but not directly providing care, e.g. WHO, technology companies and news outlets) and academic‐based sources (e.g. scientists and universities).

Figure 2 presents the division between our five stakeholder groups and the topics to reveal the stakeholder groups' usage pattern on Twitter.

The bar chart demonstrates how the majority of tweets displayed opinions, information and guidelines, as well as awards and events, while education and experience were far less frequently tweeted about. Individuals and professionals made the most tweets concerning opinions, experiences and education. In contrast, the stakeholders within organizations & companies and Academia tweeted mainly about information, news, promoting events and awards, research output and offered job vacancies.

The third‐largest topic was 'Complimenting Other'. This topic included tweets such as *'A heartwarming presentation from @#### about Person Centred Care and advocacy for women with a disability in our Maternity Service*'. This topic does not present a perception of PCC but is a performative exercise and common feature on Twitter.^17^, ^18^ Similarly, the topic 'Questions & Quoting' consisted of posts about requiring or copying information from others, which serves to display oneself as interesting rather than discuss the topic of the tweet.^20^

### 3.2 Qualitative content analysis

This section concerns the second part of the aim, which aimed to explore the content of the PCC tweets and required Twitter content that revealed a perception of PCC. Therefore, most topics did not apply, that is 'Information & Guidelines', 'Awards & Events', 'Education', 'Academic', 'Job vacancy', 'Complimenting Other', 'Questions & Quoting' and 'News'. These topics only provided information without deeper expression of the meaning of PCC for the stakeholder.

In the qualitative analysis, we used 428 tweets from the category of 'Opinions' and 'Experiences'. A split became apparent of positive and negative perceptions of PCC. This division was defined as 'Care should be person‐centred' (n = 253) and 'Challenging to realize' (n = 175). From this division emerged three sub‐themes that were analogous between the positive and negative tweets which are discussed here (see Table 2).

**TABLE 2:** Distribution of tweets for the qualitative content analysis

Tweets for analysis (n=428)	Category title	Sub‐category
Positive perceptions	Care should be person‐centred (n=253) 59%	Vision of PCC (n=127) 50%
		Facilitating PCC (n=91) 36%
		Experience of PCC (n=35) 14%
		
Negative perceptions	Challenging to realize (n=175) 41%	PCC is not that simple (n=74) 42%
		Lack of resources (n=57) 33%
		No access (n=44) 25%

### 3.3 Care should be person‐centred

This first theme, with 253 tweets (59%), was an overall positive expression of PCC and emphasized the importance of PCC in health care. The tweets in the theme of 'Care should be person‐centred' were overwhelmingly positive about this type of care and how it can change people's lives when practised. In particular, posting content on how health care should be person‐centred seemed popular and surpassed the mentions of real‐life experiences of PCC (see Figure 2).

Half of the tweets (n=127) focused on 'the vision of PCC'. These tweets represented a desire for a more PCC approach, which was often explicitly sought for groups that can be made vulnerable, such as the elderly, people with disability and the LGBT+ community.

Integrated, person‐centered care & accessible, high‐quality #community support should CAN be pillars of our health system. People regardless of their 'ability', what they've been through, where they are now ‐deserve care suited to their needs. #StateOfCare #ACEs (Organizations & Companies)

Person‐Centred Care for LGBTQI people is our #1 priority! #lgbt #lgbtqi #lgbtcare #cqc #healthcare #socialcare (Health‐care facility)

Another sub‐theme, with 91 tweets (36%), addressed the facilitation of PCC by health‐care professionals. These tweets contained references to acts of person‐centredness from the perspective of the health‐care provider. Often these tweets demonstrated elements of PCC that were possible to achieve in the context of the health‐care facility.

REPLY @#### Uniforms can be a useful visual aid for a person who needs assistance. However, I sometimes wear regular clothes when helping those who hate "white suits" ‐ so there is an argument, but it all comes down to person centred care. Being flexible and adaptable is key. (Health‐care professional)

The third sub‐theme, with 35 tweets (14%), highlighted individuals receiving PCC. These tweets expressed a sense of thankfulness that they received this type of care, as PCC is not common practice.

Took me back to my Input days.... feeling especially lucky that all my physio contacts in primary and tertiary care were exactly this. Compassionate, holistic and completely person centred. I hadn't realised this may have been exceptional at the time; 15 years ago (Individual)

@’health service' brilliant & outstanding outpatients experience today with person centred consultant rheumatologist ####. Gave time & great care to my daughter thank you (Individual)

### 3.4 PCC is challenging to realize

One hundred seventy‐five (41%) tweets had a more critical perspective on PCC. The tweets in this theme of the challenges of realizing PCC have shown the everyday difficulties with this model of care. The largest sub‐theme, with 74 tweets (42%), communicated how contradicting interests or traditions affected the possibility of providing PCC. These tweets contained content emphasizing that moving on from words is complicated.

REPLY @#### The political class (Lib Lab) don't understand what 'person‐centred care' is. They are locked into provider‐centred care systems ‐ unions will accept nothing else on the ALP (American Labour Party) side; the AMA (American Medical Association) will accept nothing else of the Coalition. It will require a 3rd pol force to achieve it. (Individual)

One‐third (n=57) of the tweets in this theme discussed the challenges when implementing PCC. These tweets underlined the effort that goes into realizing a PCC environment for patients.

@### @### @### This is really important for care providers to read.. we dont believe murals are person centred and therefore we don't use them. They are often disorienting for individuals causing unnecessary distress, as highlighted.. than you for sharing #carehomes #dementia (Health‐care facility)

@#### @### @### @### I work in Aged Care and the sad truth is we just don't have enough time to treat people as they should be. Person Centred Care models are fine but without the time and a proper amount of staff you just can't deliver it. (Health‐care professional)

The last sub‐theme pointed out the lack of access (25% and n=44) to individual PCC encounters. A number of these tweets underscored that health‐care providers said they provide PCC but that the reality was different and that patients did not actually have access to PCC.

I honestly can't believe I am about to go through another social care complaints procedure due to not adhering to the care act, or person centred care planning. I despair. Do SW (social workers) not look at the work being done by SWE (Social Work Education) (Individual)

@###### Since mum was diagnosed 9 months ago, we have not received person centred care. We have been to 8 group meetings and had a nurse allocated to us who has changed recently. We are fighting our way through trying to find help and services. Please DM if you need any details. (Individual)

## 4 DISCUSSION

This study aimed to investigate different stakeholder groups' usage patterns on Twitter and to explore the content of their PCC tweets. This study explored if there is a consensus to be found in the content of the tweets from different stakeholders that can lead to a more unified conceptualization of PCC. The stakeholders' topics showed that the majority of tweets had content regarding opinions, information and guidelines, as well as the promotion of awards and events. At the same time, education and experience were far less frequently tweeted. The perceptions of PCC in the qualitative content analysis showed a similar result. Most tweets concerned how care should be envisioned; nonetheless, there was shown to be a lack of realization of that vision, that is, how to operationalize PCC into everyday life. There is an understanding of the importance of PCC, but the information in the tweets is dominated by the motives for posting the tweets, for example coming across as interesting or update others about prior and future PCC‐related events.

The qualitative content analysis generated two main themes: 'Care should be person‐centred' and 'PCC is difficult to realize'. Most tweets in both themes concerned PCC as a model of care and emphasized that PCC should especially be available for specific groups, such as the elderly. However, this implementation is not straightforward as there are different interests and traditions in health care. As other literature has highlighted, the culture within health‐care contexts is difficult to change.^4^, ^27^, ^28^, ^29^ Previous research has shown that PCC concept developed differently in particular fields such as dementia or cancer and that the lack of a common consensus definition and concept, even with successful research, has hampered mainstream implementation.^13^ Our results are in line with these findings, presenting evidence that even Twitter feeds show that the will for PCC is there, yet the lack of real‐world experience outside of research is still very much lacking.

When the health‐care setting could provide PCC, there was overarching positivity regarding the effect it had on the patients. However, due to a lack of resources or bad experiences, PCC was not always the preferred model of care to provide. These results demonstrate a need for careful consideration of the implementation of PCC and its associated costs. Previous literature has argued that time constraints, increased workload and negative professional attitudes form barriers to providing PCC.^4^, ^30^ Instead, health‐care personnel choose to provide 'person‐centred moments' for their patients, in which the health‐care team shares humanistic values, such as mutual respect for individuals and their rights.^31^ These moments can evolve into a PCC culture, but the question remains if the ‘moments’ are enough to consider the care ‘person‐centred’. Previous research in PCC suggested that a systematic approach towards the PCC partnership, that is creating PCC all the time, not only when ‘there is time or moments for PCC’, is the most effective.^3^, ^32^

The experience of the individuals who received PCC showcased its positive effects when it is adequately operationalized. The tweets showed experiences of 'person‐centred moments' from the perspective of the patients. Other studies on patients' experiences and perceptions of PCC have shown that when professionals express thoughts unrelated to the patient's illness, this was considered a positive experience.^33^, ^34^, ^35^, ^36^ The tweets about the absence of PCC showed the need for ample opportunity to receive PCC. Negative experiences came from the patients knowing they should receive PCC but that the health‐care providers do not systematically work with it.^4^, ^33^

Twitter is an excellent platform for getting ideas across, as demonstrated by the mostly information‐sharing content of the tweets. PCC is a topic that appeared to score well on Twitter but had diverse uses. Twitter inspires more loose, distal connections between users where reciprocity becomes arbitrary.^37^ The findings exemplified this as the vast majority of the tweets concerned educating others about their knowledge and vision of PCC. The tweets in this study seemed crafted to perform as an extension of the individual, thereby surpassing a discussion of PCC to enhance their online identity. Twitter is nevertheless a widely used online policy‐ and lobbying tool, making it a critical thermometer for the current social media communication concerning PCC.

### 4.1 Methodological limitations

Although this study complements previous research on PCC, there are several limitations to Twitter‐based research. Contrary to classic data collection methods, the tweets are data‐driven, which leaves no space to ask for clarification. The Twitter API only collects the tweets with the search terms and could be snippets within conversations. Therefore, 31 tweets could not be adequately analysed for lack of context. Besides, we solely analysed tweets with the concept of ´Person’‐centred care, which may cause tweets that meant PCC but used different terms to be excluded.

The COSTCARES network provided translations of PCC in almost all European languages. However, only five translations of PCC were presented in the Twitter sample (i.e. Danish, Swedish, German, Spanish and Dutch). It occurred that non‐English tweets had not translated the concept of PCC. This lack of representation is likely due to a combination of two factors: Twitter is not equally popular across Europe, and the translations of PCC might not be applied in practice.

Lastly, the tweets were only collected for one month, and the data may, therefore, be skewed towards the events and the active Twitter users of that particular month. However, this limitation occurs with any given time frame.

## 5 CONCLUSION

This is the first study into the representation of Person‐Centred Care on Twitter. The results showed that the PCC content focused on providing information and the stakeholders' opinions rather than contributing to the PCC discussion or expressing their experiences in practice. The meaning in the tweets revealed the appeal of PCC but that it is challenging to execute in practice and implementation needs careful consideration. The overall perception is that 'quality' care cannot be provided if it is not person‐centred, but the reality seems to be that we still just talk and dream about PCC, rather than practise and experience it.

## Supporting information

Supplementary Table

## Data Availability

The data that support the findings of this study are available upon reasonable request from the corresponding author.
