# Supplementary material for: "Care is not care if it isn't person‐centred": A content analysis of how Person‐Centred Care is expressed on Twitter
Source: Health Expect. 2021 Jan 28;24(2):548–55. doi: 10.1111/hex.13199 (PMC8077091; doi:10.1111/hex.13199)
Supplement: Supplementary file 1 — Supplementary Table [file HEX-24-548-s001.docx]

Table 1. API search terms derived from the Cost Action 15222 COST CARES network

| Language | Term |
| --- | --- |
| British English | Person Centred Care |
| American English | Person centered Care |
| Bosnian/Croation | Briga sa osobom u središtu |
| Croation | Skrb usmjerena osobi |
| Ukrainian | здравствена заштита усмерена ка особи |
| Greek | Προσωποκεντρική φροντίδα |
| Bulgarian/Macedonion | грижа насочена кон лицето |
| Portuguese | Cuidados centrados na pessoa |
| Serbian | Zdravstvena zaštita usmerena ka osobi |
| Danish | Personcentreret pleje |
| Norwegian | Personcentreret omsorg |
| Danish | Personcentreret behandling |
| German | Personenzentrierte Versorgung |
| German | Personenzentrierter Ansatz |
| Hebrew | טיפול ממוקד מטופל |
| Dutch | Persoonsgerichte zorg |
| Norwegian | Personsentrert omsorg |
| Spanish | atención centrado en la persona |
| Swedish | Personcentrerad vård |
| Swedish | Personcentrerat arbetssätt |
| German | Personenzentrierte gesundheitsversorgung |
| German | personenzentrierte pflege |
| Finnish | Ihmislähtöinen hoitotyö |
| Finnish | Ihmislähtöinen hoito |
